# Personalized Approaches to Diabetic Foot Care: The Impact of Ethnic and Socioeconomic Disparities

**DOI:** 10.3390/jpm15040133

**Published:** 2025-03-29

**Authors:** Tal Shachar, Eyal Yaacobi, Roy Romem, Mohamad Fadila, Geva Sarrabia, Mor Saban, Nissim Ohana

**Affiliations:** 1 Affiliated with Faculty of Medicine and Health Sciences, Tel Aviv University, Ramat Aviv, Tel Aviv 69978, Israel; tshachar1@gmail.com (T.S.); yaacobi.eyal@gmail.com (E.Y.); roy.romem@gmail.com (R.R.); mohamad.fadila@gmail.com (M.F.); geva017@gmail.com (G.S.); 2 Nursing Department, The Stanley Steyer School of Health Professions, Faculty of Medical and Health Sciences, Tel Aviv University, Ramat Aviv, Tel Aviv 69978, Israel; morsab1608@gmail.com; 3 Department of Orthopaedic Surgery, Meir Medical Center, 59 Tchernichovsky St., Kfar Saba 4428163, Israel

**Keywords:** personalized medicine, diabetic foot ulcers, health disparities, socioeconomic status, ethnic differences, precision healthcare, healthcare equity

## Abstract

**Objective**: To explore the impact of ethnic and socioeconomic disparities on diabetic foot ulcer (DFU) care and outcomes, emphasizing the need for personalized treatment approaches tailored to diverse patient populations. **Methods**: This retrospective observational study analyzed 1409 patients hospitalized with DFUs between 2016 and 2023 at a tertiary medical center. Data extracted from electronic medical records included demographics, socioeconomic status (SES), clinical variables, and healthcare utilization. Statistical analyses included descriptive statistics, Chi-Square and Kruskal–Wallis tests, and a regularized logistic regression model to identify predictors of mortality and disparities in treatment access. **Results**: Arab patients had significantly lower SES (median score: 3.00) compared to Jewish patients (median score: 8.00), resulting in reduced healthcare access and worse clinical outcomes. Arab patients were diagnosed with diabetes at a younger age (57 years vs. 68 years for Jewish patients) and exhibited a higher body mass index (30.36 vs. 28.68, *p* < 0.05). Despite similar mortality rates between groups (21.52% vs. 22.83%, *p* = 0.65), differences in healthcare utilization were evident, particularly in younger patients (18–59 years) within the internal medicine department (*p* = 0.017). **Conclusions**: Our findings underscore the need for a personalized approach to diabetic foot care, integrating socioeconomic and demographic factors into treatment plans. Ethnic minorities with lower SES, earlier diabetes onset, and higher BMI may require tailored intervention strategies to optimize prevention, access to specialized care, and adherence to treatment. Addressing individualized patient needs through precision medicine and culturally adapted healthcare models can improve outcomes and reduce disparities in DFU management.

## 1. Introduction

Diabetic foot ulcers (DFUs) represent a major complication of diabetes mellitus, contributing to significant morbidity, prolonged disability, and increased healthcare costs worldwide [1]. These ulcers develop due to a complex interplay of neuropathy, ischemia, and foot deformities, which are further influenced by individual patient factors, including glycemic control, comorbidities, and adherence to preventive care [2,3]. However, a purely medical approach is insufficient; effective DFU management requires a patient-centered strategy that accounts for socioeconomic and ethnic disparities in healthcare access and treatment adherence [4].

With the global prevalence of diabetes projected to rise from 537 million in 2021 to 783 million by 2045, the incidence of DFUs will continue to increase [5]. It is estimated that 15–25% of individuals with diabetes will develop a DFU in their lifetime, underscoring the need for personalized risk assessment to improve prevention and treatment strategies [6].

A precision medicine approach to DFU care involves tailoring treatment plans based on patient-specific factors such as age, sex, ethnicity, and socioeconomic status (SES) [7]. Older adults (≥65 years) are particularly vulnerable due to age-related physiological changes, including diminished skin elasticity, reduced peripheral circulation, and a higher burden of comorbidities, all of which impact wound healing and response to therapy [2,7,8]. Similarly, sex-based differences have been observed, with men exhibiting a higher risk of DFU development, potentially due to differences in foot care habits, lifestyle behaviors, and the prevalence of peripheral artery disease [9,10]. These demographic variations emphasize the importance of personalized DFU treatment plans that consider individual risk factors rather than a one-size-fits-all approach.

SES further plays a critical role in DFU prevention, treatment accessibility, and outcomes [11]. Patients from lower SES backgrounds frequently encounter barriers to quality healthcare, including financial constraints, reduced access to specialized foot care services, and lower health literacy, leading to delayed diagnoses and suboptimal treatment [12,13]. By incorporating SES data into patient risk assessments, healthcare providers can develop tailored interventions such as enhanced patient education, mobile health services, and culturally adapted foot care programs to bridge these gaps and improve treatment adherence.

Beyond standard treatment protocols, personalized medicine in DFU care extends to individualized therapeutic approaches. Orthopedic interventions, including customized footwear, orthotics, and offloading devices, should be adjusted based on biomechanics, ulcer severity, and lifestyle constraints [14]. In advanced cases requiring surgery, treatment selection must consider patient-specific factors such as vascular status, wound chronicity, and the likelihood of postoperative adherence [15,16,17].

This study aims to investigate the role of sociodemographic and ethnic disparities in DFU outcomes, emphasizing the need for personalized treatment approaches. By understanding how SES, ethnicity, and demographic factors shape healthcare access and response to treatment, we can develop precision-driven interventions that optimize care and reduce disparities. This research aligns with the growing emphasis on personalized medicine as a framework for achieving more equitable and effective diabetic foot care.

## 2. Methods

### 2.1. Study Design and Setting

This retrospective archival study was conducted at a single medical center over an eight-year period, spanning from January 2016 to December 2023. The study received approval from the institutional review board (IRB) of our medical center and was performed in compliance with the ethical standards set forth in the 1964 Declaration of Helsinki and its subsequent amendments or comparable ethical guidelines. As the study was retrospective in nature and relied on anonymized data extracted from electronic medical records, the IRB granted a waiver of informed consent. Data were collected from patients’ Electronic Medical Records (EMRs), which included sociodemographic information such as age, gender, ethnicity, and residential area. Each patient’s residential area was linked to a socioeconomic (SE) cluster, as defined by the Central Bureau of Statistics (CBS). These SE clusters, ranging from 1 (lowest) to 10 (highest), are determined based on factors such as population demographics, education levels, employment status, and standard of living.

In addition to sociodemographic data, information on hospitalization was obtained, including the date of admission, body mass index (BMI), Padua score, Norton scale, Morse fall scale, date of surgery, date of discharge, and mortality status. The Padua score is used to assess the risk of venous thromboembolism (VTE) in hospitalized patients. The Norton scale evaluates the risk of developing pressure ulcers based on factors such as mobility, incontinence, and mental condition. The Morse Fall Scale estimates the likelihood of patient falls during hospitalization, incorporating variables such as history of falls, secondary diagnoses, and gait disturbances. The hospital’s orthopedic department features a specialized unit dedicated to the treatment of diabetic foot and infectious wounds, while diabetic foot ulcer patients are also managed within the internal medicine department.

The decision regarding whether a patient was hospitalized in the internal medicine ward or the orthopedic ward was made solely on a medical basis, depending on the patient’s clinical status. The policy at our institution ensured that patients admitted to the internal medicine ward received routine, daily visits from an orthopedic surgeon, who provided consultation and performed surgical procedures when necessary.

For this study, data were identified and extracted for all patients admitted with diabetic foot ulcers to either the orthopedic or internal medicine departments during the study period. Hospital admission criteria included the presence of moderate to severe infection, systemic manifestations (e.g., fever and leukocytosis), need for surgical intervention, advanced wound care, or failure of outpatient management. Admission decisions were based on the clinical evaluation of severity and multidisciplinary assessment.

### 2.2. Data Analysis

The data analysis utilized various statistical methods to examine differences in diabetes care and related outcomes across ethnic groups. Descriptive statistics were initially calculated to summarize the demographic and clinical characteristics of the study participants, including measures such as means, standard deviations, and proportions for variables like age, sex, BMI, and marital status.

To compare differences between ethnic groups, appropriate statistical tests were applied. The Chi-Square test was used for categorical variables, such as sex, death rates, and marital status, to determine whether significant differences existed in their distribution between Jewish and Arab participants. For numerical variables, including age, number of children, BMI, Padua score, Norton scale, and Morse fall scale, the Kruskal–Wallis test was employed due to the non-normal distribution of these variables, as confirmed by the Shapiro–Wilk normality test.

For analyzing healthcare utilization patterns over time, ARIMA (Auto Regressive Integrated Moving Average) modeling was applied to identify trends and patterns in the data from 2016 to 2024. The Augmented Dickey–Fuller (ADF) test was conducted to assess stationarity, while the Ljung–Box test was used to evaluate autocorrelation in the residuals, ensuring the reliability of the time-series analysis.

To identify key predictors of mortality among the study participants, a regularized logistic regression model (L2 regularization) was used. This model allowed for the assessment of the relative importance of various clinical and demographic factors, such as age, BMI, and comorbid conditions, in predicting mortality.

Disparities in socioeconomic status (SES) between Arab and Jewish diabetic patients were examined using weighted Mann–Whitney U tests, providing a non-parametric approach to compare SES distributions. Effect sizes were calculated using Cliff’s Delta and Cohen’s d to quantify the magnitude of these differences, while descriptive statistics were used to offer additional context.

Various diagnostic tests were conducted to validate the models used in the analysis, including tests for heteroskedasticity (H test) and normality of residuals (Jarque–Bera test). These tests ensured the robustness and accuracy of the findings.

The combination of these statistical techniques provided a thorough understanding of the disparities in diabetes care and outcomes among the ethnic groups studied. The significant findings underscore the need for targeted interventions to address the identified disparities.

All statistical analyses were performed using Python (version 3.9).

## 3. Result

### 3.1. Demographic and Clinical Characteristics

The study included a total of 1409 participants, comprising 1041 (73.9%) individuals identifying as Jewish and 368 (26.1%) identifying as Arab. The demographic and clinical characteristics of the participants are summarized in Table 1.

The gender distribution was similar between the groups, with females representing 37.18% of the Jewish participants and 38.6% of the Arab participants (*p* = 0.67), while males constituted 62.82% of the Jewish group and 61.4% of the Arab group. However, there was a significant difference in the average age, with Jewish participants being older (70.63 ± 16.51 years) compared to their Arab counterparts (62.52 ± 14.84 years) (*p* < 0.001). This age disparity could influence the clinical outcomes and healthcare needs observed in each group, potentially contributing to different risk profiles for complications associated with diabetic foot ulcers.

Mortality rates were comparable between the groups, with 21.52% of Jewish participants and 22.83% of Arab participants succumbing to their conditions during the study period (*p* = 0.65). The similarity in death rates suggests that, despite the differences in age and other demographic factors, mortality outcomes may be influenced by common factors affecting both groups, such as the severity of diabetic foot complications and access to care.

Regarding clinical measures, Arab participants had a significantly higher average body mass index (BMI) (30.36 ± 15.6) than Jewish participants (28.68 ± 11.36) (*p* < 0.05). This difference may reflect underlying disparities in lifestyle, dietary habits, or comorbid conditions such as obesity, which could exacerbate the risk of diabetic complications.

The clinical assessment scores did not show significant differences between the groups. The Padua score, which measures the risk of venous thromboembolism, was similar for Jewish (3.64 ± 2.27) and Arab (3.75 ± 2.28) participants (*p* = 0.51). The Norton scale, used to assess the risk of pressure ulcers, indicated no significant variation, with Jewish participants scoring 16.14 ± 3.62 and Arab participants scoring 16.71 ± 3.07 (*p* = 0.46). Similarly, the Morse fall scale, which evaluates fall risk, showed comparable results between the groups, with scores of 49.09 ± 22.8 for Jewish participants and 50.49 ± 23.78 for Arab participants (*p* = 0.14). These findings suggest that the risk factors for secondary complications related to diabetic foot ulcers were consistent across ethnic groups.

The data highlight certain sociodemographic and clinical differences that may inform targeted strategies for improving diabetic foot care. The higher BMI in Arab patients and the older age of Jewish patients could influence individualized management approaches aimed at reducing the burden of diabetic foot complications.

### 3.2. Distribution of Patients by Age Group and Ethnicity

**Figure 1:**
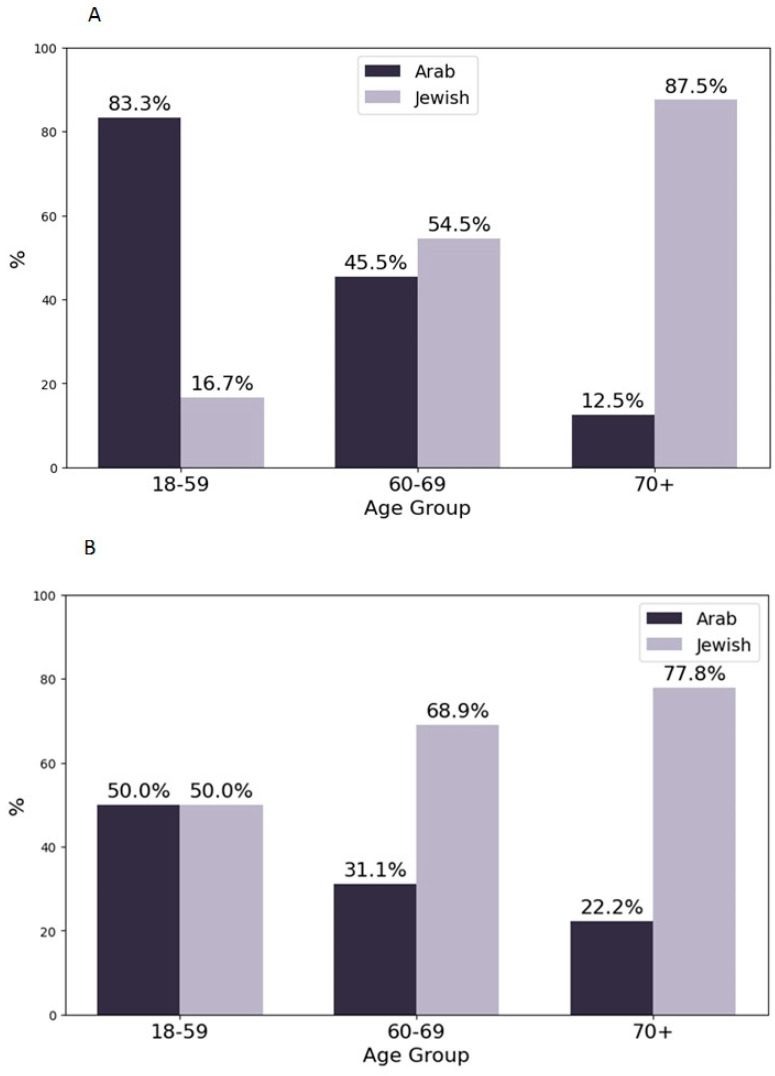
Distribution of patients undergoing surgery by age group and ethnicity in internal medicine (**A**) and orthopedics departments (**B**).

Figure 1 illustrates the distribution of patients undergoing surgery in the internal medicine and orthopedics departments, categorized by age group and ethnicity. The surgical procedures performed included debridement of necrotic tissue, drainage of abscesses, minor amputations such as toe or transmetatarsal amputations, and, in severe cases, major limb amputations. The decision for surgical intervention was guided by clinical severity and multidisciplinary evaluation.

The analysis aimed to identify whether significant differences existed in the representation of Jewish and Arab patients across various age groups in the two departments.

In the 18–59 age group, a significant difference was observed in the internal medicine department, with Jewish and Arab patients distributed unevenly (Chi-Square test: *p* = 0.017; Two-Proportion Z-Test: *p* = 0.0046). This indicates that ethnicity played a role in the likelihood of younger patients being treated in the internal medicine department. However, no significant differences were found in the orthopedics department for this age group (Chi-Square test: *p* = 0.467; Two-Proportion Z-Test: *p* = 0.381), suggesting similar proportions of Jewish and Arab patients received orthopedic care in this age range.

In the 60–69 age group, there were no significant differences in the distribution of patients by ethnicity in either department (Internal Medicine: Chi-Square test: *p* = 0.480, Two-Proportion Z-Test: *p* = 0.297; Orthopedics: Chi-Square test: *p* = 0.957, Two-Proportion Z-Test: *p* = 0.827). This finding suggests that, for middle-aged to older adults, the proportion of Jewish and Arab patients undergoing surgery was consistent across both departments, potentially reflecting similar healthcare needs or referral practices.

For patients aged 70 and above, the analysis showed no significant differences in either the internal medicine or orthopedics departments (internal medicine: Chi-Square test: *p* = 1.0, Two-Proportion Z-Test: *p* = 0.782; orthopedics: Chi-Square test: *p* = 0.412, Two-Proportion Z-Test: *p* = 0.327). This indicates that the oldest age group had comparable representation of Jewish and Arab patients in both departments, suggesting that advanced age may have outweighed ethnicity in determining the choice of surgical care setting.

These findings highlight that age, in conjunction with ethnicity, may influence the allocation of surgical patients to different departments, particularly for younger individuals in internal medicine. The lack of significant differences in older age groups could be due to the common healthcare needs associated with advanced age across both ethnicities.

### 3.3. Feature Importance in the Prediction of Death

**Figure 2:**
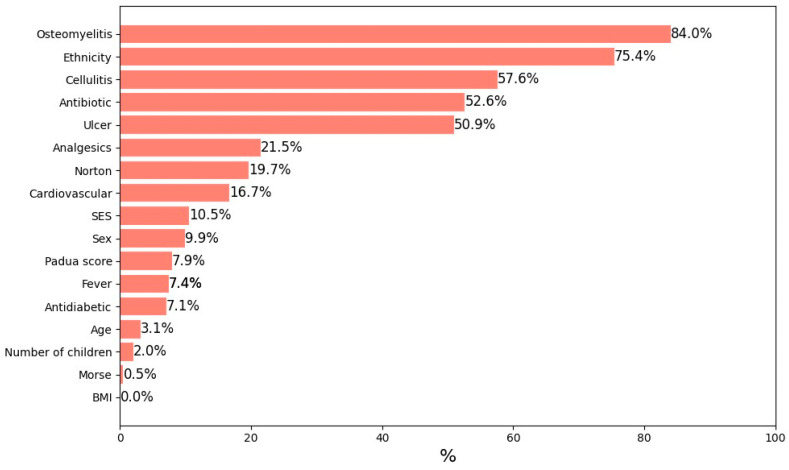
Feature importance in predicting mortality. The figure shows the most influential factors in predicting death, including age, BMI, and comorbidities, as identified by a regularized logistic regression model (L2).

Figure 2 illustrates the feature importance of predicting mortality, as determined by a regularized logistic regression model (L2 regularization) applied to the entire study population.

Figure 2 displays the results of a regularized logistic regression model, where the coefficients represent the relative importance of various predictors of mortality. Age had the highest model coefficient, indicating its dominant role as a mortality risk factor. This statistical result reflects the greater vulnerability of older adults with diabetic foot ulcers, who are more likely to suffer from comorbidities and physiological decline contributing to poor outcomes. BMI was the second most influential feature, with higher BMI values associated with an elevated risk of death, possibly due to the increased likelihood of obesity-related complications. Additionally, the presence of comorbid conditions, particularly hypertension and cardiovascular disease, significantly contributed to mortality risk. These chronic conditions can exacerbate the severity of diabetic foot complications and impact overall health outcomes.

Other factors, such as gender, socioeconomic status, and hospitalization duration, also contributed to the model’s predictive accuracy, although their influence was less pronounced compared to age, BMI, and comorbidities. The inclusion of a broad range of clinical and demographic variables enabled the model to achieve high accuracy and reliability in identifying patients at higher risk of death. This underscores the importance of a comprehensive approach to patient assessment, incorporating multiple factors to improve management strategies and optimize prognosis (Figure 2).

### 3.4. Healthcare Utilization Patterns

**Figure 3:**
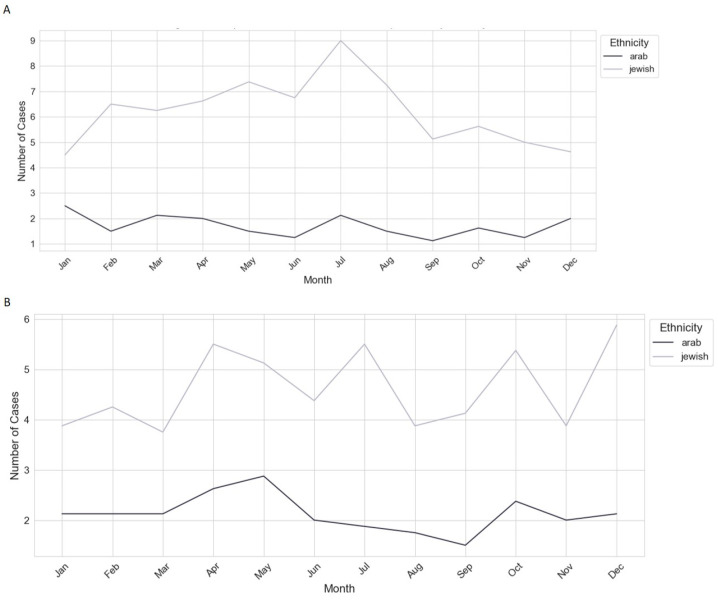
Ethnic comparison of monthly case averages in internal medicine (**A**) and orthopedics (**B**), 2016–2024. The figure shows higher overall healthcare utilization among the Jewish population and greater volatility in trends for the Arab population, with notable peaks and dips.

Figure 3 presents the ethnic comparison of monthly case averages in internal medicine (A) and orthopedics (B) from 2016 to 2024, analyzing healthcare utilization patterns among Arab and Jewish populations.

The time series analysis revealed distinct trends, with both groups exhibiting stationary patterns and identical model coefficients, suggesting similar underlying factors influencing healthcare utilization.

However, the overall utilization was higher among the Jewish population (trend range: 0.4–1.0) compared to the Arab population (trend range: 0.1–0.7), indicating greater healthcare engagement or access.

The trends for the Arab population showed more pronounced volatility, with notable peaks occurring in 2018, 2019, 2022, and 2023, possibly reflecting periods of increased health service demand due to specific events or seasonal factors. In contrast, the Jewish population’s trends were more stable, with significant dips observed in 2018, 2021, and 2022, suggesting fluctuations in healthcare access or service delivery during these years.

Diagnostic tests confirmed the absence of significant autocorrelation in the residuals, though potential heteroskedasticity and non-normality of residuals were noted, indicating variability in error terms across the series. The presence of a significant moving average component in both models suggests that recent random events have a stronger influence on current healthcare utilization than do longer-term historical patterns.

These findings highlight disparities in healthcare utilization between Arab and Jewish populations, warranting further investigation into the socioeconomic, cultural, and systemic factors that may drive these differences. Understanding these factors is crucial for addressing healthcare inequities and optimizing resource allocation (Figure 3).

### 3.5. Socioeconomic Status Disparities

**Figure 4:**
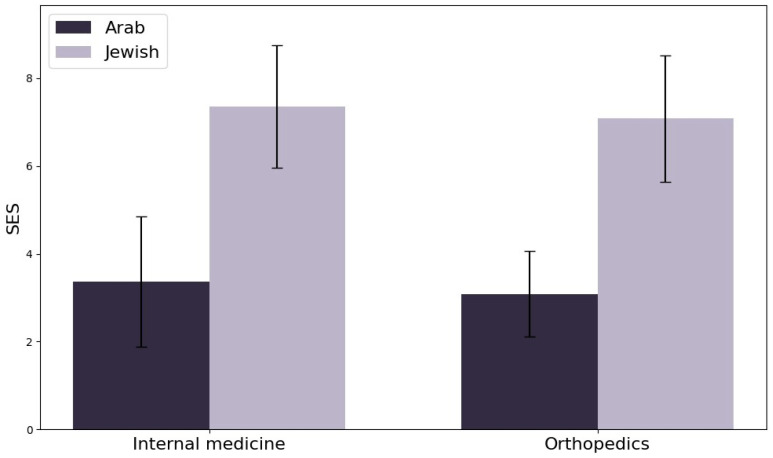
Socioeconomic status disparities among Arab and Jewish diabetic patients in internal medicine and orthopedics. The figure shows significant SES differences, with Jewish patients having notably higher SES scores than Arab patients in both departments.

Figure 4 presents an in-depth analysis of socioeconomic status (SES) among Arab and Jewish diabetic patients hospitalized in the internal medicine and orthopedics departments. The analysis reveals substantial SES disparities between the two groups, indicating significant socioeconomic inequality.

Statistical tests confirmed these differences, with weighted Mann–Whitney U tests showing highly significant results (*p* < 0.0001) in both departments. The effect sizes were large, with Cliff’s Delta values of −0.86 (internal medicine) and −0.92 (orthopedics), and corresponding Cohen’s d values of −2.81 and −3.06. These values indicate that the SES distribution is considerably lower among Arab patients compared to Jewish patients, with the vast majority of Jewish patients having higher SES scores.

Descriptive statistics highlight the extent of the gap, with median SES scores of 3.00 for Arab patients and 8.00 for Jewish patients in both departments. This consistency across departments suggests that systemic factors, such as differences in access to education, employment opportunities, and healthcare resources, may be driving these disparities. The magnitude and persistence of these SES differences underscore the need for targeted interventions to address the root causes of socioeconomic inequality and their impact on health outcomes. Strategies may include policies aimed at improving access to quality healthcare, educational programs, and economic support for disadvantaged populations.

Further investigation into the social determinants of health is essential to reduce disparities and improve outcomes for lower SES groups (Figure 4).

## 4. Discussion

Diabetes disproportionately affects ethnic minority groups, including migrants from Turkey, Morocco, South Asia, and sub-Saharan Africa, who experience higher prevalence rates and more severe complications compared to the general population [18,19]. These disparities are shaped by a combination of genetic predisposition, socioeconomic challenges, and lifestyle changes following migration [20]. Notably, ethnic minorities are often diagnosed with diabetes at a younger age, leading to an extended disease burden and a higher likelihood of complications such as diabetic foot ulcers (DFUs) [20]. This early onset poses unique challenges for personalized diabetes care, as treatment approaches must consider cultural, linguistic, and socioeconomic barriers that influence health literacy, treatment adherence, and clinical outcomes [18,20]. It is important to note that Israel’s healthcare system provides universal coverage to all citizens, including both Jewish and Arab populations, through publicly funded health maintenance organizations (HMOs). Access to essential medical services is uniformly available, regardless of income or ethnic background. Even in universal healthcare systems, systemic inequalities persist, reinforcing the need for tailored, patient-centered interventions that address both medical and social determinants of health [19,20].

A key finding in our study is the significant disparity in socioeconomic status (SES) between ethnic groups, with Jewish patients demonstrating higher SES (median score: 8.00) compared to Arab patients (median score: 3.00). We also acknowledge that the Jewish population in Israel is ethnically heterogeneous, encompassing subgroups such as Ashkenazi, Sephardi, Mizrahi, and Ethiopian Jews. Some of these groups may share cultural or genetic similarities with Arab populations, which could influence health behaviors and outcomes. However, due to limitations in our data structure, we were unable to stratify Jewish participants into these subcategories. These socioeconomic differences directly impact healthcare access, treatment adherence, and clinical outcomes, as lower SES is often associated with financial barriers, reduced access to preventive care, and poorer overall health outcomes [21,22]. Patients with limited financial resources may struggle to afford proper foot care, delay seeking medical attention, or face logistical barriers in reaching specialized diabetic foot clinics. Personalized medicine approaches must integrate SES-related risk factors to develop targeted strategies that improve access to preventive care, enhance patient education, and facilitate multidisciplinary treatment models [23].

### 4.1. Age, Healthcare Utilization, and Disease Complexity

Our analysis of healthcare utilization patterns across different age groups revealed notable departmental differences. Among younger patients (18–59 years), Arab patients were more likely to be hospitalized in the Internal Medicine Department (*p* = 0.017), while no significant differences were observed in the Orthopedics Department (*p* = 0.467). This suggests that younger patients present with more complex, multi-systemic conditions requiring broader medical management rather than isolated DFU-related orthopedic care. These findings align with personalized medicine principles, emphasizing the need for age-specific treatment pathways that address not only DFU management but also underlying metabolic and cardiovascular conditions [24].

A key distinction in our findings is that patients admitted to Internal Medicine were often hospitalized for systemic disease rather than for DFU management alone, whereas those in Orthopedics were primarily treated for DFUs. This supports the integration of interdisciplinary care models where internal medicine specialists, endocrinologists, and vascular surgeons collaborate to optimize DFU treatment alongside comprehensive metabolic care. The earlier onset of diabetes in Arab patients (57 years vs. 68 years for Jewish patients) underscores the need for early intervention programs tailored to at-risk populations [24].

Interestingly, no significant ethnic differences were observed in Orthopedic Department admissions, suggesting that when surgical intervention is required, both groups access orthopedic services at similar rates. This finding may reflect a more standardized, protocol-driven approach in surgical DFU management, reinforcing the importance of early risk stratification and referral systems to ensure equitable access to preventive care before surgical intervention becomes necessary.

### 4.2. Personalized Interventions for Obesity-Associated DFU Risk

Obesity was a notable differentiating factor between the groups, with Arab patients exhibiting a significantly higher BMI (30.36 vs. 28.68, *p* < 0.05). Obesity is a well-documented risk factor for DFU development and poor healing outcomes, as it contributes to chronic inflammation, insulin resistance, and increased mechanical stress on the lower extremities [20,22]. Studies have shown that Arab women, in particular, have higher obesity rates (52.2% vs. 31.4% in Jewish women), often due to cultural and environmental factors limiting engagement in physical activity [11,25].

Personalized diabetes care must extend beyond glycemic control to include culturally adapted lifestyle interventions that address dietary habits, physical activity barriers, and behavioral modifications. Healthcare providers should consider community-based prevention programs, gender-specific interventions, and digital health solutions to support long-term lifestyle changes in high-risk populations [25].

Despite the BMI differences, no significant differences were observed in Padua scores, suggesting that venous thromboembolism risk may be influenced by additional lifestyle or clinical factors beyond BMI alone. This highlights the importance of individualized risk assessment strategies, where clinicians account for activity levels, cardiovascular risk profiles, and adherence to thromboprophylaxis protocols when developing personalized DFU management plans.

### 4.3. Mortality Predictors and Risk Stratification

Our analysis identified age as the strongest predictor of mortality, consistent with existing literature demonstrating that older adults face higher mortality risks due to cumulative comorbidities, physiological decline, and prolonged disease duration. This reinforces the need for age-adapted clinical pathways that emphasize aggressive prevention, early screening, and proactive DFU management in elderly populations.

Beyond age, BMI and comorbid conditions such as hypertension and cardiovascular disease were key predictors of mortality. Higher BMI is associated with metabolic dysfunction, while cardiovascular disease exacerbates DFU progression, increasing amputation and mortality risk [20,22]. Personalized medicine strategies should incorporate machine learning-based risk models to identify high-risk patients early and implement targeted interventions, such as intensive wound surveillance, advanced wound care therapies, and optimized cardiovascular management.

A study by Shashar et al. [26] demonstrated that embedding an internal medicine specialist within an orthopedic department improved 30-day mortality trends by enhancing the comprehensive management of high-risk DFU patients. This supports the integration of multidisciplinary teams that combine metabolic, vascular, and wound care expertise to optimize outcomes in high-risk populations.

### 4.4. Healthcare Utilization Patterns and Personalized Resource Allocation

Our findings revealed significant disparities in healthcare utilization, with Jewish patients exhibiting higher overall hospital utilization (trend range: 0.4–1.0) compared to Arab patients (0.1–0.7). The greater volatility in Arab patient utilization, with peaks in 2018, 2019, 2022, and 2023, suggests fluctuations in healthcare access potentially influenced by policy changes, economic instability, or shifts in healthcare service availability. In contrast, Jewish patients exhibited more stable trends, likely reflecting better healthcare engagement and continuity of care.

These patterns highlight the need for personalized healthcare policies that target underutilized and underserved populations. Strategies such as mobile wound care units, telemedicine consultations, and culturally adapted patient education programs could help bridge these access gaps and ensure equitable DFU care delivery.

This study has several limitations that should be considered when interpreting the findings. First, as a retrospective, single-center analysis, the generalizability of our results may be limited. The reliance on electronic medical records (EMRs) introduces potential data inconsistencies and missing information, particularly in variables such as socioeconomic status (SES) and comorbidities, which may not have been uniformly documented. Moreover, our socioeconomic status (SES) data were derived from the Central Bureau of Statistics (CBS)-defined geographic clusters, which provide a proxy for socioeconomic positioning. However, individual-level data on income and employment status were not available in our dataset, limiting our ability to perform a more granular socioeconomic analysis. Additionally, the study did not account for prior care pathways, such as private or religious-affiliated healthcare services, which could influence disease presentation or referral patterns. These data were not available in our electronic medical record system and may represent a potential source of confounding. Future studies should consider prospective, multicenter designs to validate these findings across broader populations and healthcare settings.

Additionally, while this study identifies significant ethnic disparities in healthcare utilization and clinical outcomes, it does not fully explore the underlying cultural, behavioral, or systemic factors that contribute to these differences. Factors such as language barriers, health literacy, healthcare-seeking behaviors, and implicit biases in medical decision-making could play a critical role in shaping disparities and warrant further investigation. A qualitative or mixed-methods approach incorporating patient perspectives and healthcare provider insights could offer a deeper understanding of these influences.

Another limitation is that this study focuses on short-term outcomes and does not include long-term follow-up data to assess chronic disease progression, recurrent hospitalizations, or amputation rates beyond the study period. Understanding how personalized interventions impact long-term diabetic foot care remains a crucial area for future research. Additionally, detailed clinical data on critical limb ischemia and standardized ulcer severity classifications such as the WIFI (Wound, Ischemia, and foot Infection) score were not uniformly available in the electronic medical records and were therefore not included in our analysis. Future prospective studies should incorporate these vascular and wound severity parameters to allow for more precise stratification and individualized treatment approaches.

Finally, the study employed a per-protocol analysis rather than an intention-to-treat approach, which may overestimate the effectiveness of the intervention among adherent patients while not fully capturing real-world adherence challenges. Moreover, while we performed logistic regression to assess predictors of mortality, multivariate adjustment for treatment disparities was not included due to the absence of a single dominant outcome parameter. This limits our ability to isolate the independent effects of ethnicity and socioeconomic status on treatment outcomes. In addition, our dataset did not include information on the ethnicity of healthcare providers involved in patient care. Although our institution employs both Jewish and Arab healthcare professionals, we cannot exclude the possibility of implicit or systemic biases influencing clinical decisions and treatment dynamics. Future studies should consider intention-to-treat analyses, incorporate comprehensive multivariable modeling, and evaluate the potential role of caregiver-related factors in order to assess the feasibility and sustainability of personalized DFU management strategies in diverse clinical settings.

Despite these limitations, our findings provide important insights into ethnic and socioeconomic disparities in diabetic foot care and emphasize the need for personalized, patient-centered interventions. Future research should aim to develop precision-driven healthcare models that integrate sociodemographic, genetic, and behavioral factors to improve outcomes and reduce disparities in DFU management.

## 5. Conclusions and Future Directions

This study reinforces the importance of personalized approaches to DFU management, integrating socioeconomic, demographic, and clinical factors into individualized treatment plans. The disparities observed highlight the need for proactive, patient-centered strategies that address the unique challenges faced by high-risk populations. Future research should explore AI-driven risk prediction models, tailored lifestyle interventions, and culturally competent healthcare frameworks to further advance precision medicine in DFU prevention and treatment.

These findings also highlight the pressing need for increased investment in public health infrastructure, preventive foot care services, and targeted funding strategies to address healthcare inequalities in diabetic populations.

## Figures and Tables

**Table 1:** Demographic and clinical characteristics of study participants by ethnicity.

	Jewish	Arab	*p*-Value
Total (%)	1041 (73.9)	368 (26.12)	
Sex—female	387 (37.18)	142 (38.6)	0.67
Sex—male	654 (62.82)	226 (61.4)	
Age	70.63 ± 16.51	62.52 ± 14.84	<0.001
Deth	224 (21.52)	84 (22.83)	0.65
BMI	28.68 ± 11.36	30.36 ± 15.6	<0.05
Pauda score	3.64 ± 2.27	3.75 ± 2.28	0.51
Norton	16.14 ± 3.62	16.71 ± 3.07	0.46
Morse	49.09 ± 22.8	50.49 ± 23.78	0.14
No. of children	2.71 ± 2.06	4.3 ± 2.83	<0.001
Marital status:			
Married	549 (52.74)	252 (68.48)	0.001
Divorce	126 (12.1)	14 (3.8)	
Widower	239 (22.63)	64 (17.39)	
Separated	3 (0.29)	1 (0.27)	
Single	111 (10.66)	35 (9.51)	

## Data Availability

The data that support the findings of this study are available from the corresponding author upon reasonable request. Due to ethical and privacy considerations, the data are not publicly available.
